# Hepatitis B or C viral infection and the risk of cervical cancer

**DOI:** 10.1186/s13027-022-00466-8

**Published:** 2022-11-01

**Authors:** Chuanfang Luo, Shuhui Yu, Jinping Zhang, Xingrao Wu, Zhongyan Dou, Zheng Li, E. Yang, Lan Zhang

**Affiliations:** 1 Department of Radiation Oncology, The Third Affiliated Hospital of Kunming Medical University (Yunnan Cancer Hospital, Yunnan Cancer Center), 519 Kunzhou Road, Kunming, 650118 China; 2 Department of Medical Administration, The Third Affiliated Hospital of Kunming Medical University (Yunnan Cancer Hospital, Yunnan Cancer Center), 519 Kunzhou Road, Kunming, 650118 China; 3 Department of Gynecologic Oncology, The Third Affiliated Hospital of Kunming Medical University (Yunnan Cancer Hospital, Yunnan Cancer Center), 519 Kunzhou Road, Kunming, 650118 China; 4 Department of Pathology, The Third Affiliated Hospital of Kunming Medical University (Yunnan Cancer Hospital, Yunnan Cancer Center), 519 Kunzhou Road, Kunming, 650118 China

## Abstract

**Background:**

The present study aimed to evaluate the effects of hepatitis B virus (HBV) or hepatitis C virus (HCV) infection on the risk of cervical cancer.

**Methods:**

We conducted a case–control study including 838 cervical cancer cases and 838 benign disease controls matched for age, ethnicity, and place of birth. Venous blood was tested for HBV and HCV serological markers. Multiple odds ratios (OR) and corresponding 95% confidence intervals (CI) for cervical cancer were estimated using logistic regression. HBV antigens were examined using immunohistochemical staining.

**Results:**

Anti-HCV was positive in 10 cases (1.2%) and 0 controls (0%). Cases had higher percentage of chronic HBV infection (HBsAg-positive/anti-HBc-positive) and prior HBV infection (HBsAg-negative/anti-HBc-positive) than controls (6.3% vs 4.4%; 11.6% vs 7.3%). Both chronic HBV infection (OR 1.6; 95% CI 1.0–2.4) and prior HBV infection (OR 1.7; 95% CI 1.2–2.4) were associated with cervical cancer in univariate logistic regression analyses. In subgroup analysis among HPV-positive patients, the association between chronic HBV infection and cervical cancer disappeared (OR 1.2; 95% CI 0.4–3.4); while in subgroup among patients younger than 50 years, the association remained significant with adjustment for HPV infection and parity (adjusted OR 2.1; 95% CI 1.0–4.4). HBsAg and HBcAg were detected in 8% and 12% of cervical cancer cases who had seropositive HBsAg, respectively. Compared with the benign controls, individuals with both HBsAg and HPV positive had an increased risk of cervical cancer (adjusted OR 67.1; 95% CI 23.4–192.7).

**Conclusions:**

HBV infection was associated with cervical cancer in patients with age younger than 50 years. Further prospective studies are needed to confirm this relationship.

**Supplementary Information:**

The online version contains supplementary material available at 10.1186/s13027-022-00466-8.

## Introduction

Cervical cancer is the fourth most common cancer and the fourth leading cause of cancer death in women worldwide. An estimated 604,000 new cases of the disease and 342,000 cancer-related deaths were reported in 2020 [1]. Chronic infection by oncogenic types of human papillomavirus (HPV) caused most cervical cancers. Other risk factors include early age of sexual debut, multiple sexual partners, multiple full-term pregnancies, history of sexually-transmitted infection, immunosuppression, such as human immunodeficiency virus (HIV) infection or after organ transplantation, and smoking [2–5]. Notably, infection with some other virus, such as Epstein-Barr virus (EBV), might contribute to the development of cervical cancer [6]. We hypothesized that hepatitis virus infection might also lead to a greater risk of cervical cancer.

Hepatitis B virus (HBV) and hepatitis C virus (HCV) infection are global health problems. Approximately 2 billion individuals have prior infection of HBV and approximately 248 million people are chronic carriers worldwide [7]. China is one of the highest prevalence areas. HCV affects more than 71 million individuals worldwide and China accounts for more than 14% of the global prevalence [8]. HBV or HCV are major causes of chronic hepatitis infection and are potentially oncogenic viruses. The virus infects not only hepatocytes, but also lymphocytes, pancreatic cells, biliary tract cells, and kidney cells, thus making a relationship with extrahepatic cancer biologically plausible [9–12]. Several epidemiological studies suggested a notable association between HBV infection and the risk of extrahepatic cancers, including non-Hodgkin lymphoma [13], nasopharyngeal carcinoma [14], gastric cancer [15], biliary tract cancer [16], pancreatic cancer [17], and endometrial carcinoma [18]. Chronic HCV infection is generally accepted to increase the risk of non-Hodgkin lymphoma and intrahepatic cholangiocarcinoma [19]. The presence of HBV in vaginal secretions [20] and semen [21] makes it biologically plausible that HBV infects the cervix epithelium and participates in the carcinogenesis of genital cancer.

A few previous studies evaluated the role of HBV infection in cervical cancer, but with conflicting results. A case–control study from Korea reported that cervical cancer was significantly related to HBV infection, with a relative risk of approximately 1.49 [22]. Data from the Sun Yat-Sen University Cancer Center also found cervical cancer to be HBV-related [adjusted odds ratio (AOR) 1.22; 95% confidence interval (CI) 1.05–1.42] [23]. Siu et al. [24] reported the HBV carrier rate was significantly higher in both the cervical cancer and the precancerous lesion group compared with that in the control group. However, other studies did not observe associations between HBV infection and cervical cancer [25–28]. To the best of our knowledge, none of these studies made any adjustment for known risk factors, including HPV infection, multiple full-term pregnancies, smoking; or potential risk factors, including alcohol consumption [29], family history of cancer [30],and body mass index (BMI) [31] in the analyses. Interactions between HBV and HPV have never been investigated. Data on the effect of HCV infection on risk of cervical cancer are lacking. Thus, we carried out this hospital-based case–control study to investigate the role of HBV and HCV infection in the development of cervical cancer.

## Methods

### Study participants

**Fig. 1:**
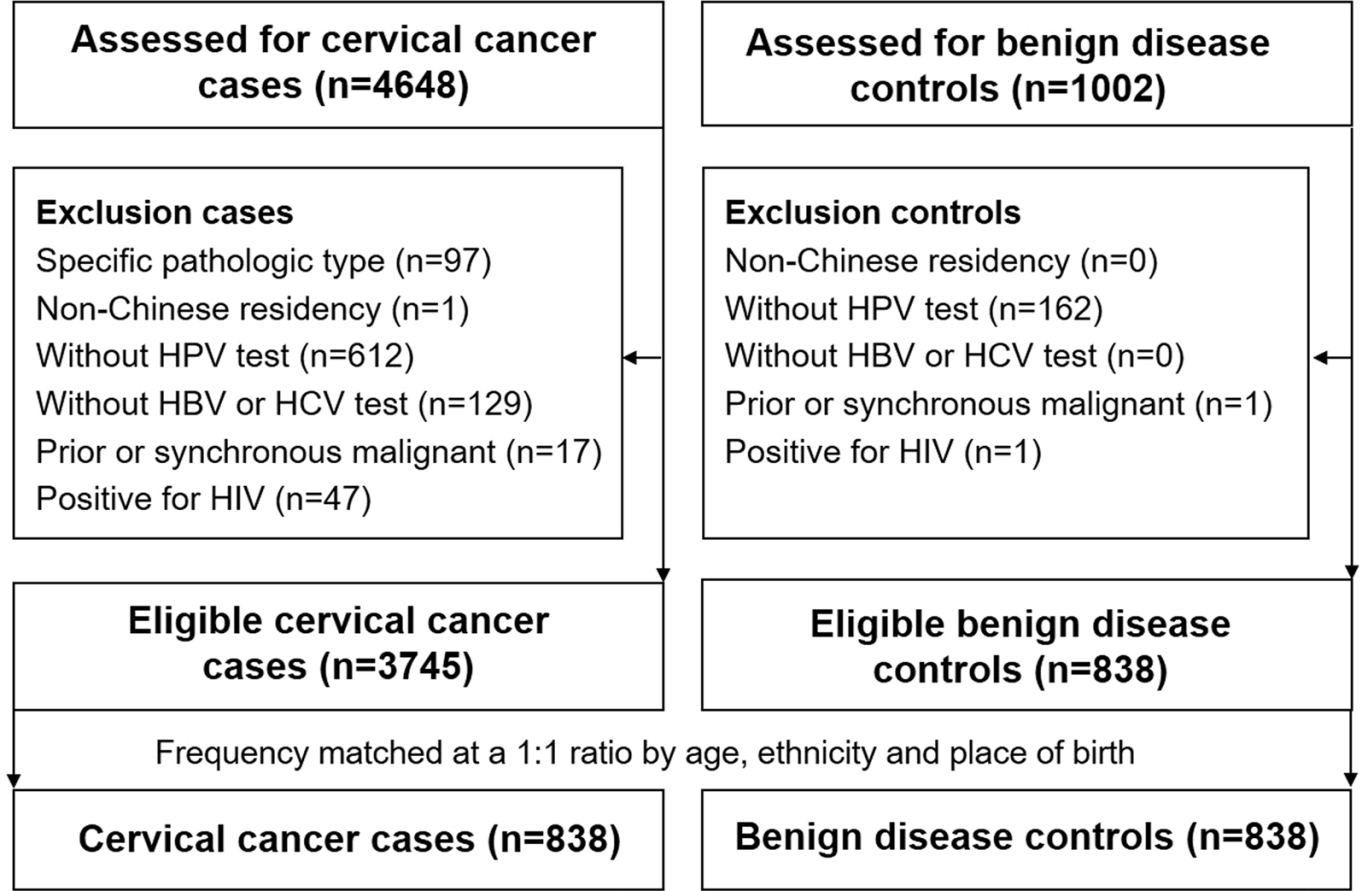
Patient flow chart. HBV, hepatitis B virus; HCV, hepatitis C virus; HPV, human papillomavirus

This study was a hospital-based, case–control study conducted at the Third Affiliated Hospital of Kunming Medical University. Patients with cervical cancer (International Classification of Diseases version 10, ICD-10 C53) and patients with benign gynecologic diseases (ICD-10 D25-28, N70-73, N80-85), as controls, were both newly diagnosed and treated from January 2015 to December 2017. A total of 4648 patients with cervical cancer and 1002 benign disease controls were identified consecutively during the study period. The exclusion criteria for cervical cancer cases were as follows: (1) without a blood test for HBV and HCV infection before treatment; (2) with HIV infection; (3) previous or synchronous malignant tumors at another organ site; (4) a specific pathological type other than squamous cell carcinoma, adenocarcinoma and adenosquamous carcinoma, such as cervical neuroendocrine carcinoma, carcinosarcoma, adenosarcoma, lymphoma, or melanoma; (5) without a test result for HPV infection at initial diagnosis; (6) non-Chinese residency. The controls comprised patients who were pathologically confirmed to have benign gynecologic diseases that were considered to be unrelated to HBV infection, mainly including uterine leiomyoma, endometriosis, mature teratoma, ovarian cystadenoma and cysts. The exclusion criteria for controls were the same as cases, except for the cancer diagnosis. Finally, there were 3748 qualified participants among the cervical cancer cases and 838 qualified participants among the benign disease controls. The list of pathologic diagnoses of benign disease is shown in Additional file 1: Table S1. To balance the baseline characteristics between the cases and controls, cases and controls were frequency matched at a ratio of 1:1 by age, ethnicity, and place of birth. 838 cases were selected for the controls to build a 1:1 case–control study (Fig. 1). Relevant information, such as cigarette smoking, alcohol drinking, family history of cancer, number of full-term pregnancies, and BMI was collected from detailed medical records reviews for both cases and controls.

The study was conducted in accordance with the Declaration of Helsinki and was approved by the Ethics Committee of Yunnan Cancer Hospital (NO. KYLX2022033). Individual consent for this retrospective analysis was waived.

### Serological assays

Venous blood was collected from both cases and controls before treatment. Serum was separated and examined for the presence of HCV antibodies (anti-HCV), HBV serological markers (including hepatitis B surface antigen (HBsAg), hepatitis B surface antigen (anti-HBs), and hepatitis B e antigen (HBeAg)), antibodies to the hepatitis B e antigen (anti-HBe), antibodies to the hepatitis B core antigen (anti-HBc), and antibodies to HIV, using enzyme-linked immunosorbent assays (WANTAI BioPharm, Beijing, China). Quality control for the measurements was performed in accordance with the protocols provided by the manufacturer.

### Clinical significance of hepatitis virus infection-related antigens and antibodies

The serological markers of HBV included HBsAg, anti-HBs, HBeAg, anti-HBe, and anti-HBc. HBsAg is a hallmark of infection, including chronic HBV infection and inactive HBsAg carrier state. Anti-HBs positivity represents acquired immunity to HBV infection, and might result from both current or prior HBV infection and vaccination. HBeAg and anti-HBe were used to show viral replication and infectivity. Anti-HBc appears 1–2 weeks after the appearance of HBsAg and is a serum marker for current or prior HBV infection [32]. HBV infection status was divided into three categories according to the status of HBsAg and anti-HBc. Individuals that were both HBsAg-negative and anti-HBc-negative were defined as never exposed to HBV; those that were HBsAg-positive and anti-HBc-positive were defined as chronic carriers of HBV; those that were HBsAg-negative and anti-HBc-positive were defined as having past exposure to HBV [17]. Individuals who were positive for anti-HCV antibodies were defined as having chronic HCV infection [33].

### HPV-DNA test

For patients from both the case and control groups, before a biopsy and pelvic examination, the surface of their cervix or vaginal canal were gently rubbed several times for DNA collection using a brush. Either a Digene Hybrid Capture 2 (HC2) High-Risk HPV DNA Test (Qiagen corporation, Hilden, Germany) or a quantitative real-time polymerase chain reaction (qPCR)-based test (BioPerfectus, Jiangsu, China) was used to evaluate the presence of HPV DNA. The HC2 test detects the presence of 13 high-risk HPV types (16/18/31/33/35/39/45/51/52/56/58/59/68). The qPCR test detected 18 high-risk HPV types (16/18/31/33/35/39/45/51/52/56/58/59/68/53/66/73/26/82).

### Immunohistochemical staining for HBsAg and HBcAg in cervical cancer tissues

The formalin-fixed, paraffin-embedded tissues of 50 seropositive HBsAg patients were cut into 4 mm sections. The tissue sections were deparaffinized and incubated to block the endogenous peroxides in 0.3% H_2_O_2_ for 10 min. Antigen retrieval was performed by treating heating sections in 10 mmol/L citric acid buffer in an autoclave for 10 min. Sections were then incubated overnight at 4 °C with antibodies against HBsAg (ZM-0122; ZSGB-BIO, Beijing, China) and antibodies against HBcAg (ZA-0121; ZSGB-BIO). Horseradish peroxidase-conjugated secondary antibodies (Kit-5010; MXB Biotechnologies, Fuzhou, China) were used as detection reagents. The reaction products were visualized using 3,3′-diaminobenzidine as the chromogen and were finally counterstained using hematoxylin. The immunohistochemical detection of each marker was visualized under a Leica CMS GmbH Image Viewer (Leica, Wetzlar, Germany). Immunohistochemically stained slides were viewed independently by two pathologists. Any positive reaction for HBsAg and HBcAg, irrespective of percentage of reactive cells, was recorded as positive.

### Statistical analysis

The statistical analyses were performed using SPSS 23.0 statistical software (IBM Corp., Armonk, NY, USA). A chi-squared test was used to compare the differences in baseline characteristics between the cases and controls. Binary logistic regression analysis was used to analyze the associations between cervical cancer and the characteristics of HCV infection, HBV antibodies and antigens, HPV infection, family history of cancer, smoking, alcohol drinking, BMI [underweight (< 18.5), normal (18.5–25), overweight (25–30), obese (> 30)], number of full-term pregnancies (0, 1, 2, 3, > 4)). Variables significant in the univariate analyses and baseline characteristics including age, ethnicity, and place of birth, were included for multivariate analyses. Multivariate logistic regression analysis was used to estimate the adjusted odds ratio (AORs) and 95% confidence interval (CI). The HBV serological markers and the three HBV infection states might have a collinear relationship; therefore, we included the HBV serological markers and the integrated HBV infection states separately in to the multivariate analyses. Tests for interaction were calculated using a multiplicative logistic regression model. A two-tailed *P*-value of < 0.05 was set as the criterion for statistical significance.

## Results

### Baseline characteristics of the cervical cancer cases and benign disease controls

The baseline characteristics for cervical cancer cases and controls are presented in Table 1. Patients were divided into three groups according to their age: < 40 years old, 40–60 years old, and > 60 years old. The numbers of patients in the three groups were 248 (29.6%), 527 (62.9%), 63 (7.5%) in the cases, and 237 (28.3%), 538 (64.2%) and 63 (7.5%) in the controls, respectively. There was a predominance of Han ethnicity among the cases (83.9%, n = 703), and the matched controls had a similar distribution of ethnicity with 719 (85.8%) Han patients. More than 97% of the patients from both cases and controls were born in Yunnan and neighboring provinces. There were no significant differences between cases and controls in terms of age, ethnicity, and place of birth (*P* = 0.834, *P* = 0.276, *P* = 0.44 respectively, Table 1).

**Table 1:** Characteristics of study population

Variable	Cases		Controls		*P*
	(n = 838)		(n = 838)		
	No	%	No	%	
Age, years					0.834
< 40	248	29.6	237	28.3	
40–60	527	62.9	538	64.2	
> 60	63	7.5	63	7.5	
Ethnicity					0.276
Han	703	83.9	719	85.8	
Non-Han	135	16.1	119	14.2	
Place of birth					0.440
Yunnan and neighboring province^a^	819	97.7	814	97.1	
Other provinces in China	19	2.3	24	2.9	

^a^Guizhou and Sichuan Province

*P* value was calculated using the chi-square test

### The associations of hepatitis virus with cervical cancer in the general study population

After adjusting for age, ethnicity, and place of birth, as well as mutually adjusting for each other, both HPV infection (AOR 57.5; 95% CI 41.8–79.2) and multiple full-term pregnancies (n = 2; AOR 4.1; 95% CI 2.2–7.8) (n = 3; AOR 7.0; 95% CI 3.3–14.7) (n > 3; AOR 4.1; 95% CI 1.8–9.3) were associated with an increased risk of cervical cancer when comparing the patients with cervical cancer to the benign disease controls (Table 2). Other factors previously reported to increase the risk of cervical cancer, including smoking [5], family history of cancer [30], alcohol drinking [29], and BMI [31], appeared to have no relationship with the risk of cervical cancer in the present study (Table 2).

**Table 2:** The associations of HPV, family history of cancer, cigarette smoking, alcohol drinking, BMI, No. full-term pregnancies, HCV, HBV infection status with the cervical cancer risk

Variable	Cases		Controls		Univariable			Multivariable		
	(n = 838)		(n = 838)							
	No	%	No	%	OR	95%CI	*P*	AOR	95%CI	*P*
HPV										
HPV-negative	141	15.5	768	91.6		1(reference)			1(reference)	
HPV-positive	697	84.5	70	8.4	55.2	40.6–75.1	** < 0.001**	57.5	41.8–79.2	** < 0.001**
Family history of cancer										
No	828	98.8	819	97.7		1(reference)				
Yes	10	1.2	19	2.3	0.5	0.2–1.1	0.106			
Cigarette smoking										
No	834	99.5	837	99.9		1(reference)				
Yes	4	0.5	1	0.1	4	0.4–36.6	0.214			
Alcohol drinking										
No	836	99.8	838	100		1(reference)				
Yes	2	0.2	0	0	–	–	–			
BMI										
< 18.5	78	9.3	67	8		1(reference)				
18.5–25	587	70	573	68.4	0.9	0.6–1.2	0.438			
25–30	150	17.9	163	19.5	0.8	0.5–1.2	0.215			
> 30	23	2.7	35	4.2	0.6	0.3–1.0	0.061			
No.full-term pregnancies										
0	40	4.8	93	11.1		1(reference)			1(reference)	
1	243	29	366	43.7	1.9	1.2–2.8	**0.004**	1.7	0.9–3.2	0.091
2	343	40.9	263	31.4	3.9	2.5–6.0	** < 0.001**	4.1	2.2–7.8	** < 0.001**
3	116	13.8	63	7.5	5.7	3.4–9.6	** < 0.001**	7	3.3–14.7	** < 0.001**
> 3	96	11.5	53	6.3	6.4	3.7–11.1	** < 0.001**	4.1	1.8–9.3	**0.001**
HCV markers										
Anti-HCV-negative	828	98.8	838	100		1(reference)				
Anti-HCV-positive	10	1.2	0	0	–	–	–			
HBV infection status										
HBsAg-negative/anti-HBc-negative^a^	686	81.9	740	88.3		1(reference)			1(reference)	
HBsAg-positive/anti-HBc-positive^b^	53	6.3	37	4.4	1.6	1.0–2.4	**0.041**	1.3	0.7–2.6	0.402
HBsAg-negative/anti-HBc-positive^c^	97	11.6	61	7.3	1.7	1.2–2.4	**0.001**	1.6	0.9–2.6	0.083

*P*-values in bold denoted statistical significance for *P* ＜0.05

Multivariate analyses were adjusted by age (as a continous variable), ethnicity (Han/non-Han), birth of place (Yunnan and neighboring province/Other provinces in China), HPV (negative/positive), and No. full-term pregnancies (0/1/2/3/ > 3)

HPV, human papillomavirus; BMI, Body Mass Index; HCV, hepatitis C virus; HBV, hepatitis B virus; HBsAg, hepatitis B surface antigen; anti-HBc, hepatitis B core antibody; OR, odds ratio; AOR, adjusted odds ratio; CI: confidence interval.

^a^Never exposed

^b^Chronic carrier of HBV

^c^Past exposure to HBV

The prevalence of HCV was higher among patients with cervical cancer (1.2%, 10/838) than among the benign disease controls (0%, 0/838) (Table 2). For the HBV serological markers, 55 (6.6%) patients were detected to have seropositive HBsAg among the cases, whereas there were 37 (4.4%) participants with seropositive HBsAg among the controls. The number of patients with seropositive HBeAg were 10 (1.2%) and 2 (0.2%) among the cases and controls, respectively. For anti-HBc-positivity, there were 150 (17.9%) and 98 (11.7%) patients in the cases and controls, respectively. Individuals with HBsAg-positivity (OR 1.5; 95% CI 1.0–2.4), HBeAg-positivity (OR 5.1; 95% CI 1.1–23.5), and anti-HBc-positivity (OR 1.7; 95% CI 1.3–2.2) were all associated with a higher risk of cervical cancer in the univariable logistic regression analyses (Additional file 2: Table S2). The associations remained significant for individuals with chronic (HBsAg-positive/anti-HBc-positive) (OR 1.6; 95% CI 1.0–2.4) or previous (HBsAg-negative/anti-HBc-positive) (OR 1.7; 95% CI 1.2–2.4) HBV infection (Table 2). Two cervical cancer patients in cases with rare HBV infection status (HBsAg-positive/anti-HBc-negative) were not shown. In the multivariate logistic regress analysis, however, the associations between HBV infection status or serological markers and cervical cancer were not significant after adjustment for age, ethnicity, place of birth, HPV infection, and multiple full-term pregnancies (Table 2; Additional file 2: Table S2).

### Subgroup analyses of patients with HPV-positive

HBV is a sexually transmitted disease that might be link to high-risk sexual behavior and subsequent HPV acquisition. It is unclear whether the association of HBV infection and cervical cancer is merely an artifact of confounding by HPV or a causal association. Therefore, we performed stratified analysis in patients with HPV-positive. Results showed that neither chronic HBV infection (OR 1.2; 95% CI 0.4–3.4) nor prior HBV infection (OR 1.1; 95% CI 0.5–3.4) was associated with cervical cancer (Table 3; Additional file 3: Table S3), suggesting HBV infection was not a risk factor for cancer development once HPV infected.

**Table 3:** The subgroup analyses for patients with HPV-positive: univariable logistic regression analysis

Variable	Cases		Controls		Univariable		
	(n = 697)		(n = 70)				
	No	%	No	%	OR	95%CI	*P*
HBV infection status							
HBsAg-negative/Anti-HBc-negative^a^	572	82.1	58	82.9		1(reference)	
HBsAg-positive/Anti-HBc-positive^b^	45	6.5	4	5.7	1.2	0.4–3.4	0.753
HBsAg-negative/Anti-HBc-positive^c^	79	11.3	8	11.4	1.1	0.5–3.4	0.873

HBV, hepatitis B virus; HBsAg, hepatitis B surface antigen; anti-HBc, hepatitis B core antibody; OR, odds ratio; CI: confidence interval

^a^Never exposed

^b^Chronic carrier of HBV

^c^Past exposure to HBV

### Subgroup analyses of patients aged younger than 50 years

Previous studies reported that HBV infection is associated with younger age at diagnosis [23]. We then explored relationship between HBV and cervical cancer stratified by age. Participants were divided into younger (< 50) and older (≥ 50) groups according to the median diagnostic age of all 4648 patients with cervical cancer. In patients aged younger than 50 years, after adjusting for age, ethnicity, place of birth, HPV infection, and number of full-term pregnancies, a modest but significant association between chronic HBV infection and cervical cancer was observed (AOR 2.1; 95% CI 1.0–4.4) (Table 4; Additional file 4: Table S4).

**Table 4:** The subgroup analyses for patients aged younger than 50 years: univariable and multivariable logistic regression analyses

Variable	Cases		Controls		Univariable			Multivariable		
	(n = 620)		(n = 616)							
	No	%	No	%	OR	95%CI	*P*	AOR	95%CI	*P*
HBV infection status										
HBsAg-negative/anti-HBc-negative^a^	502	81	543	88		1(reference)			1(reference)	
HBsAg-positive/anti-HBc-positive^b^	73	12	27	4.4	1.7	1.1–2.9	**0.03**	2.1	1.0–4.4	**0.046**
HBsAg-negative/anti-HBc-positive^c^	43	6.9	46	7.5	1.7	1.2–2.6	**0.005**	1.5	0.8–2.8	0.194

*P*-values in bold denoted statistical significance for *P* ＜0.05

HBV, hepatitis B virus; HBsAg, hepatitis B surface antigen; anti-HBc, hepatitis B core antibody; OR, odds ratio; AOR, adjusted odds ratio; CI: confidence interval. Multivariate analyses were adjusted by age (as a continous variable), ethnicity (Han/non-Han), birth of place (Yunnan and neighboring province/Other provinces in China), HPV (negative/positive), and No.full-term pregnancies(0/1/2/3/ > 3)

^a^Never exposed

^b^Chronic carrier of HBV

^c^Past exposure to HBV

### Synergistic effects of HBV and HPV infection

To explore the synergistic effects of HBV and HPV infection, we performed stratified analyses. By separating the participants into four subgroups according to HBsAg (negative/positive) and HPV (negative/positive) status, we found that the AOR appeared to be greatest among patients with both HBsAg and HPV positivity (AOR 67.1; 95% CI 23.4–192.7), although no significant interaction was observed between HBsAg and HPV (*P* = 0.636) (Table 5). We also separated the population into another four subgroups according to anti-HBc (negative/positive) and HPV (negative/positive) status. Similarly, the AOR appeared to be greater among individuals with both anti-HBc and HPV positivity (AOR 59.5; 95% CI 31.6–112.2), although no significant interaction was observed between anti-HBc and HPV (*P* = 0.159) (Table 5).

**Table 5:** Modification effect of HPV infection between the association of HBV and the risk of cervical cancer with stratified analyses

Variable	Cases		Controls		Univariate analyses			Multivariate analyses		
	(n = 838)		(n = 838)							
	No	%	No	%	OR	95%CI	*P*	AOR	95%CI	*P*
HBsAg and HPV										
HBsAg-negative/HPV-negative	132	16	735	88		1(reference)			1(reference)	
HBsAg-negative/HPV-positive	651	78	66	7.9	54.9	40.1–75.2	** < 0.001**	58.5	42.0–81.3	** < 0.001**
HBsAg-positive/HPV-negative	9	1.1	33	3.9	1.5	0.7–3.2	0.281	1.6	0.7–3.5	0.252
HBsAg-positive/HPV-positive	46	5.5	4	0.5	64.0	22.7–180.9	** < 0.001**	67.1	23.4–192.7	** < 0.001**
* P*_interaction_							0.69			0.636
Anti-HBc and HPV										
Anti-HBc-negative/HPV-negative	115	14	682	81		1(reference)			1(reference)	
Anti-HBc-negative/HPV-positive	573	68	58	6.9	58.6	41.9–81.9	** < 0.001**	59.1	42.0–83.3	** < 0.001**
Anti-HBc-positive/HPV-negative	26	3.1	86	10	1.8	1.1–2.9	**0.017**	1.8	1.1–3.0	**0.018**
Anti-HBc-positive/HPV-positive	124	15	12	1.4	61.3	32.8–114.4	** < 0.001**	59.5	31.6–112.2	** < 0.001**
* P*_interaction_							0.192			0.159

*P*-values in bold denoted statistical significance for *P* ＜0.05

Multivariate analyses were adjusted by age (as a continous variable), ethnicity (Han/non-Han), birth of place (Yunnan and neighboring province/Other provinces in China) and No. full-term pregnancies(0/1/2/3/ > 3)

HBV, hepatitis B virus; HBsAg, hepatitis B surface antigen; anti-HBc, hepatitis B core antibody; OR, odds ratio; AOR, adjusted odds ratio; CI: confidence interval

### Detection of HBsAg and HBcAg in cervical cancer tissues

**Fig. 2:**
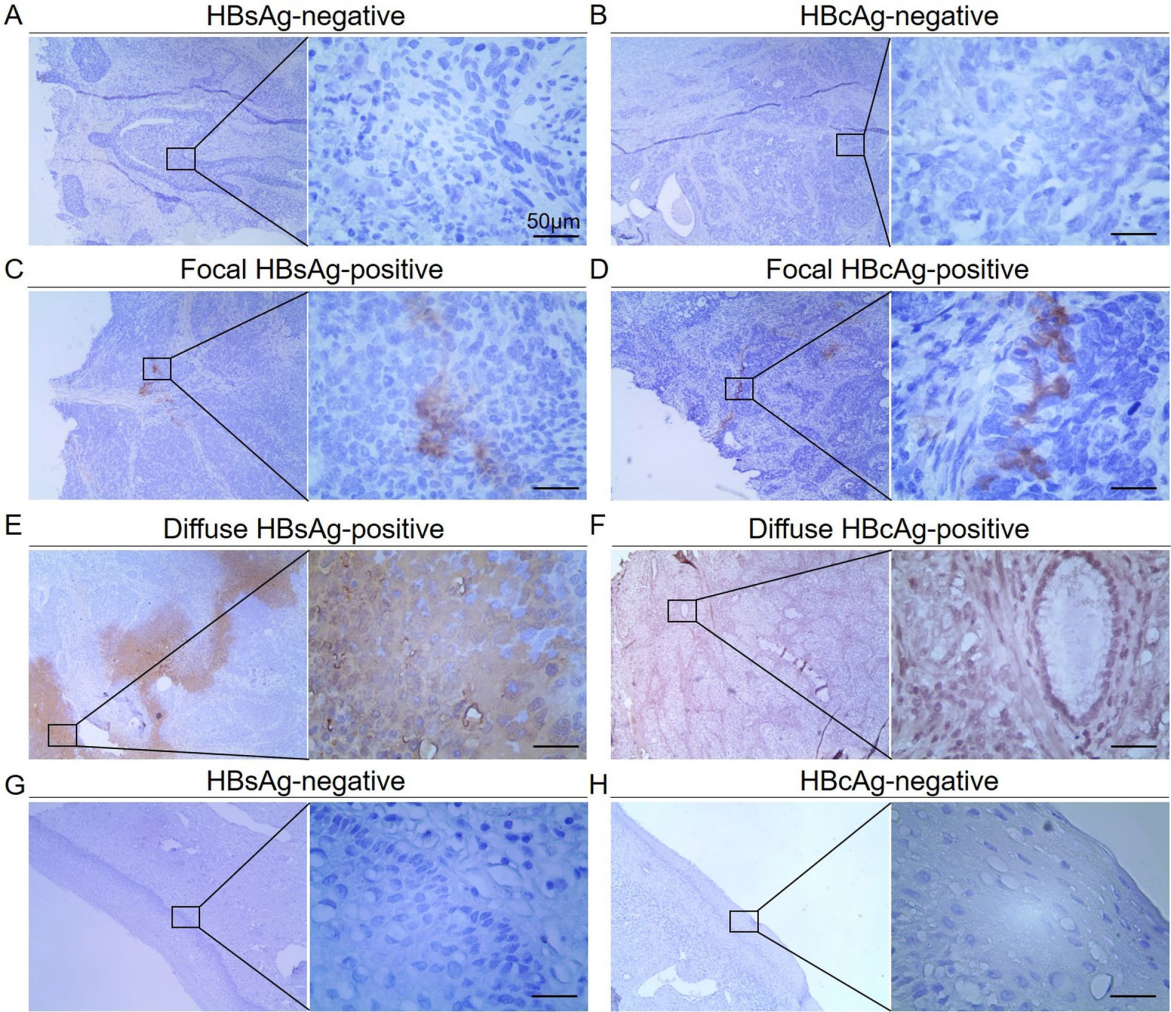
Immunohistochemistry assay for HBV antigens. Representative images of **A** HBsAg-negative, **B** HBcAg-negative, **C** focal HBsAg-positive, **D** focal HBcAg-positive, **E** diffuse HBsAg-positive, **F** diffuse HBcAg-positive staining in cervical cancer tissues. Representative images of **G** HBsAg-negative, **H** HBcAg-negative staining in adjacent normal cervical epithelium. Bar = 50 μm

Previous studies demonstrated the existence of HBV antigens in extrahepatic tissues [17, 23] to explain the relationship between HBV infection and cancer development. As a sexually transmitted virus, HBV was biologic plausibility to infect cervical tissues. We collected 50 formalin fixed, paraffin embedded sections of cervical cancer tissues with positive serum HBsAg to detect HBV antigens using immunohistochemical staining. The majority of those patients showed negative staining for both HBsAg (46/50, 92%) and HBcAg (44/50, 88%). HBsAg was detectable in four cases (8%) and HBcAg was detectable in six cases (12%). Focal HBsAg staining was seen in two cases (4%) and focal HBcAg staining was observed in three cases (6%). Diffuse cytoplasmic HBsAg staining was seen in two cases (4%) and diffuse HBcAg staining was seen in three cases (6%). Both HBsAg and HBcAg were detectable in one case. 41 of these 50 cervical cancer samples had adjacent normal epithelium in the formalin-paraffin embedded tissues. All of the adjacent normal epithelium showed negative staining for HBsAg (0/41, 0%) and HBcAg (0/41, 0%). Representative immunohistochemical staining of HBsAg and HBcAg in cervical cancer tissues are shown in Fig. 2.

## Discussion

Cervical cancer is the most commonly diagnosed gynecologic malignancy in developing countries. Infection with HPV is the most well-established cause of cervical cancer. Women with high-risk sexual behaviours had elevated risk of developing cervical neoplasia after the adjustment for HPV infection, suggesting other sexual transmitted agents may play an etiologic role [34]. HBV is a known sexual transmitted agent affecting millions of people worldwide. Some epidemiological studies suggested that HBV infection could influence the risk of cervix malignancy [22, 23], while others showed conflicting results [25–28]. Whether and to what extent HBV infection increases the risk of cervical cancer is unclear. In the present study, we first reported that HBV infection might be associated with a modest risk of cervical cancer in patients aged younger than 50 years.

HBV and HPV are both sexual transmitted viruses, the increase of HPV infection rate could be accompanied by the increase of HBV. It is important to exclude the influence of HPV infection by stratified analysis. On the stratum of HPV-positive cases and controls, no significant association was found between HBV infection and cervical cancer, suggesting HBV infection was not a risk factor once HPV infected. However, we could not completely exclude the biologic role of HBV in cervical cancer development. For there were only 70 HPV-positive patients in control group, the limited sample size and relatively low HBV-infection rate may decrease the study power. Interestingly, we found a significant association between chronic HBV infection and cervical cancer in participants aged younger than 50 years with adjustment for HPV and full-term pregnancies, suggesting HBV infection might be an HPV-independent risk factor for the younger cervical cancer patients.

HBV infection causes dysfunction of innate and adaptive immune responses [35], and the incidence of genital HPV infection might increase when immunosuppression or immunodeficiency occurs. A previous study showed that HPV and HBV could integrate into the same sites in the human genome [36]. This evidence makes it possible that HBV could interact with HPV to induce and promote the pathogenesis of cervical cancer. We therefore examined the interactions between HPV and HBV infection. The results showed that there was no statistically significant interaction of HPV with HBV infection serological markers HBsAg and anti-HBc, although the AORs appeared greater in participants with HPV infection and seropositive HBsAg. Given the limited numbers of participants in each subgroup of the present study, a real interaction between HBV and HPV infection needs to be confirmed in a future large-scale prospective study.

The potential mechanism underlying the association between HBV infection and cervical cancer development is unknown. The impact and mechanism of HBV infection in the development of hepatocellular carcinoma has been thoroughly studied. HBV DNA integrating into the genome of the host directly induces chromosomal instability and causes alterations of host gene expression and signaling pathways [37]. Previous studies found evidence of hepatitis virus infection and replication in extrahepatic tissues, suggesting HBV infection might increase the risk of extrahepatic cancer via a similar mechanism to that in HBV-related hepatocellular carcinoma [23]. The detection of HBV DNA in vaginal secretions [20] and semen [21] made us suspect that HBV might directly infect the cervix epithelium and promote the pathogenesis of malignancy. In this study, we detected HBV antigens in cervical cancer tissues, demonstrating viral replication in cervix tissues. However, the positive rates of HBV antigens in patients with positive serum HBsAg were extremely low, suggesting that direct viral infection in the cervix epithelium might be not the primary factor responsible for the association between HBV and cervical cancer development.

It is unclear why the impact of HBV infection was only significant in patients younger than 50 years. One possible explanation for this result may be the complex sexual behaviour in the younger individuals. High-risk behaviour is an HPV-independent risk factor for cervical neoplasia. The younger individuals are prone to have high-risk sexual behaviors than the older age group, which leads more chances to develop into cancer. The other reason we suspected is that the oncogenic role of HBV infection might age dependent. HBV infection is demonstrated to be associated with younger median age at diagnosis multiple cancers [23]. HBsAg induces a large number of cytokines, including transforming growth factor β and interleukin-10 [38], which might increase susceptibility to develop cancer [39]. A previous study reported that HBsAg levels declined with age in HBV infected patients [40]. We hypothesized that the oncogenic action of HBV might be more obvious in younger patients who have higher levels of HBsAg than older patients who have decreased levels of HBsAg. Future studies are required to confirm the role of HBV infection in younger patients and determine its detailed mechanisms.

A few limitations of the present study should be addressed. First, without a healthy control group, we could not rule out the possibility of selection bias resulting from the use of controls with various kinds of benign diseases; this might have resulted in overestimation or underestimation of the prevalence of HBV infection among control participants. However, it is less likely that our finding of the positive relationship between HBV and cervical cancer is confounded by selection bias for the following reasons: (1) The prevalence of HBsAg among our controls was consistent with the overall prevalence in China of 4.57% [41] and the HPV infection rate was in line with that reported 8.3% in Yunnan province [42], indicating that the control group was representative of the source population in terms of HBV and HPV infection; (2) demographic and major risk factors of cervical cancer were adjusted; (3) controls represent the study hospital from which cervical cancer patients were selected. Second, the number of HPV-positive controls in the subgroup analysis is too few. The small sample size and the relatively low HBV infection rate render this study more susceptible to inherent selection bias. Whether HBV infection is an independent risk factor for cervical cancer or work as a risk modifier of HPV exposure need to be investigated in further studies. Third, we lack a known time sequence between the infection of HBV and HPV. The causal link between HBV with HPV infection is controversial because of the marked correlation between the two infections and sexual activity. Longitudinal cohort studies with information of HPV and HBV infection status included are needed to confirm the association of HBV with risk of cervical cancer.

## Conclusion

In conclusion, based on a hospital-based case–control study of cervical cancer in Southwestern China, we found that HBV infection had a significant association with cervical cancer in patients aged younger than 50 years. Preventing HBV infection might have a beneficial effect to reduce the risk of cervical cancer. The association between HBV or HCV infection and cervical cancer should be confirmed by further prospective studies.

## Supplementary Information

**Additional file 1. Table S1**: Pathologic diagnoses and ICD-10 codes of patients in the control group.

**Additional file 2. Table S2**: The associations of HBV serologic markers with cervical cancer risk: univariable and multivariable logistic regression analyses.

**Additional file 3. Table S3**: The subgroup analyses for patients HPV-positive: univariable logistic regression analysis.

**Additional file 4. Table S4**: The subgroup analyses for patients aged younger than 50 years: univariable and multivariable logistic regression analyses.

## Data Availability

The data used to support the findings of this study are available from the Research Ethical Committees of Yunnan Cancer Hospital (Contact via Email: ynzlyyll@163.com).

## Abbreviations

AOR: Adjusted odds ratio
EBV: Epstein-Barr virus
HBV: Hepatitis B virus
HBsAg: Hepatitis B surface antigen
anti-HBs: Antibodies against hepatitis B surface antigen
HBeAg: Hepatitis B envelope antigen
anti-HBe: Antibodies against hepatitis B envelope antigen
HBcAg: Hepatitis B core antigen
anti-HBc: Antibodies against hepatitis B core antigen
HCV: Hepatitis C virus
HIV: Human immunodeficiency virus
HPV: Human papillomavirus
CI: Confidence intervals
OR: Odds ratio
ICD: International classification of disease
